# Comparative Analysis of the Mitochondrial Genome of *Galatheanthemum* sp. MT-2020 (Actiniaria Galatheanthemidae) From a Depth of 9,462 m at the Mariana Trench

**DOI:** 10.3389/fgene.2022.854009

**Published:** 2022-06-08

**Authors:** Mengke Shi, Li Qi, Li-Sheng He

**Affiliations:** ^1^ Institute of Deep-sea Science and Engineering, Chinese Academy of Sciences, Sanya, China; ^2^ University of Chinese Academy of Sciences, Beijing, China

**Keywords:** galatheanthemum, hadal zone, extreme environment, adaptation, mitochondrial genome

## Abstract

The hadal zone, which represents the deepest marine habitat on Earth (6,000–11,000 m), is a harsh environment mainly characterized by extremely high hydrostatic pressure, and this habitat is believed to have a high degree of endemism. The deep-sea anemone family Galatheanthemidae comprises two valid species exclusively from the hadal; however, no other information about this family is currently available. In the present study, a sea anemone was collected from a depth of 9,462 m at the Mariana Trench and was defined as *Galatheanthemum* sp. MT-2020 (Actiniaria Galatheanthemidae). The mitochondrial genome of *Galatheanthemum* sp. MT-2020 was circular, was 16,633 bp in length, and contained two ribosomal RNA genes, 13 protein-coding genes and two transfer RNA genes. The order of the genes of *Galatheanthemum* sp. MT-2020 was identical to that of the majority of the species of the order Actiniaria. The value of the AT-skew was the lowest in the whole mitochondrial genome, with a positive GC skew value for the atp8 gene, while other species, except *Antholoba achates*, had the negative values of the GC skew. *Galatheanthemum* sp. MT-2020 was clustered with another abyssal species, *Paraphelliactis xishaensis,* in the phylogenetic tree, and these species diverged in the early Jurassic approximately 200 Mya from the shallow-sea species. The usage ratio of valine, which is one of the five amino acids with the strongest barophilic properties, in the mitochondrial genomes of the two abyssal species was significantly higher than that in other species with habitats above the depth of 3,000 m. The ω (dN/dS) ratio of the genomes was 2.45-fold higher than that of the shallow-sea species, indicating a slower evolutionary rate. Overall, the present study is the first to provide a complete mitogenome of sea anemones from the hadal and reveal some characteristics that may be associated with adaptation to an extreme environment.

## Introduction

The hadal zone is primarily composed of ocean trenches, represents the deepest marine habitat on Earth (6,000–11,000 m) and is one of the last great frontiers in marine science, accounting for 45% of the total ocean depth range (Jamieson, 2015). The hadal zone is a harsh environment characterised by extremely high hydrostatic pressure, accumulation of food along the trench axes, constant darkness, slightly increased bottom temperature and geographical isolation, which is believed to have caused a high degree of endemism in this habitat, making the hadal zone unique and one of the least understood habitats on Earth (Jamieson, 2011).

The sea anemone (Actiniaria) is one of the most diverse and successful members of the anthozoan subclass Hexacorallia (Reft and Daly, 2012) and is distributed across diverse benthic marine habitats, even in the deep water (Ammons and Daly, 2008) and polar areas (Fautin et al., 2013). Moreover, actinians are dominant in the hadal fauna (Wolff, 1970). Galatheanthemidae is one of the families of sea anemones and is commonly known as the deep-sea species. Previous studies have shown that Galatheanthemidae is a monogeneric family comprising only two currently validated species (Cairns et al., 2007) exclusively present at the depths between 5,900 and 10,700 m (Wolff, 1959; Jamieson, 2011); however, no other information about these species is available. In October 2020, a sea anemone was obtained from a depth of 9,462 m in the Mariana Trench by the “Fen Dou Zhe” manned submersible and defined as *Galatheanthemum* sp. MT-2020. The morphology of species of the *Galatheanthemum* genus is usually presented as a long column of approximately 25 cm with simply flattened ends and with a cuticular surface possibly filled with a layer of mucous containing many spirocysts; the species lack the disc-like structure, and this morphology is a typical characteristic of *Galatheanthemum*.

Mitochondrial DNA (mtDNA) sequences have been widely used in the studies of comparative and evolutionary genomics, species identification, population genetics, phylogeography and phylogenetic relationships (Gissi et al., 2008). Mitochondrial genomes of only fifteen families of Actiniaria have been reported to date despite numerous known species. All reported mitochondrial genomes of sea anemone are circular, between 16 and 20 kb in length, and include 13 energy pathway protein-encoding genes. Presumably due to more primitive evolutionary position of anemones, their mtDNA possesses some distinctive features, such as the presence of only two tRNA genes, unlike commonly observed 22 tRNAs, and frequent occurrence of nonconventional and optional genes. Insertion-like ORFA and the presence of complex group I introns representat a widespread unconventional mitochondrial genes in sea anemones (Beagley et al., 1996; Flot and Tillier, 2007; Beagley and Wolstenholme, 2013; Osigus et al., 2013; Emblem et al., 2014; Chi et al., 2018; Johansen and Emblem, 2020; Johansen et al., 2021). Among all available mitochondrial genomes of Actiniaria, only the genomes of *Bolocera* sp. from a depth of 1,106 m and *Paraphelliactis xishaensis* from a depth above 3,230 m have been reported, and there is no reference information from the hadal zone. In the present study, the mitogenome of *Galatheanthemum* sp. MT-2020 was sequenced, assembled and analysed together with previously published mitogenomes to infer phylogenetic relationships between these genomes, describe their mtDNA characteristics and calculate their divergence times.

## Materials and Methods

### Sample Collection, DNA Extraction and Sequencing

The specimen used in the present study was sampled from the Mariana Trench (E142°11.4152′, N11°19.4990′) at a depth of 9,462 m by the manned submersible “Fen Dou Zhe” in October 2020. Once onboard, the specimen was fixed in 75% ethanol and stored at 4 °C. The muscle of the specimen was isolated, and DNA was extracted using a genomic DNA kit (Tiangen Co., Beijing, China) according to the manufacturer’s instructions. DNA library was constructed and sequenced by BGI, Qingdao. Sequencing was performed in the paired-end mode by an Illumina HiSeq platform. Adapters and sequences with a quality score below 15 (Phred33 format) were removed from the raw reads using Trimmomatic 0.36. The clean data were assembled by SPAdes v3.10.1 (k-mer = 21–77) and NOVOPlasty v.3.8.3 (Dierckxsens et al., 2017) with default settings. To identify the contigs of mitochondrial origin, a BLAST search was conducted using an available mitogenome of *Bolocera* sp. BZ-2016 as a reference (GenBank accession number KU507297). We used seqMan of the DNASTAR software package (http://www.dnastar.com) to detect the sequences of the repeats at the beginning and end of the extracted contigs, and the results showed that the sequence was circular. Then, we identified cox1 of the extracted contigs as the seed sequence, and the mitogenome of this species was processed by NOVOPlasty using the seed extension algorithm to complete the assembly. The results of NOVOPlasty confirmed that the mitogenome was circular.

### Annotation and Analysis

The mitochondrial genome was annotated using the MITOS web server (http://mitos2.bioinf.uni-leipzig.de/index.py) (Donath et al., 2019) and subsequent manual annotation. Briefly, the boundaries of 13 PCGs (Protein-coding genes) and two ribosomal RNA (rRNA) genes were determined by comparison with the homologous genes of other Actiniaria species. The new mitogenome sequence has been deposited in GenBank under the accession number OL912950. The transfer RNA genes (tRNAs) and their secondary structures were predicted using tRNAscan-SE 1.21 (Lowe and Eddy, 1997) and RNA structure Web (https://rna.urmc.rochester.edu/RNAstructureWeb/). Nucleotide composition was computed using the MEGAX (Kumar et al., 2018) program. The skew values of AT and GC were calculated according to the following equations: AT skew = (A− T)/(A+ T) and GC skew = (G − C)/(G + C), respectively, where A, T, G and C corresponded to the percentages of the corresponding bases. Codon usage counts and the relative synonymous codon usage (RSCU) values were calculated using MEGAX based on the table of the coelenterate mitochondrial genetic code. The nucleotide diversity (Pi) of 13 PCGs and two rRNAs of the mitochondrial genome was calculated using DnaSP 6.0 software with a sliding window of 100 bp and a step size of 25 bp. The amino acids of 13 PCGs were classified into the nonpolar, polar uncharged and charged residues. The percentages of each type of amino acid were calculated and compared with other sea anemones with complete mitochondrial protein-coding regions using a statistical *t* test.

### Construction of Phylogenetic Tree

To investigate the phylogenetic relationships between the one from a 9,462 m depth and other reported species, two phylogenetic trees were constructed in this study. Besides the one from 9,462 m depth, the first tree including 128 sea anemone species (Rodriguez et al., 2014) was constructed by three genes (12S, 16S, and cox3) using the Maximum Likelihood approach, and showed in Supplementary Figure S1. Another tree including 28 sea anemone species (Supplementary Table S1) was constructed by the nucleotide sequences of the mitochondrial 13 protein-coding genes and showed in Figure 6A. *Chrysopathes formosa* and *Siderastrea radians* were used as the outgroups. The nucleotide sequences of 13 protein-coding genes were used as the dataset to build the maximum likelihood (ML) and Bayesian inference (BI) phylogenetic trees. MAFFT was used to align the nucleotide sequences. Conserved regions were identified using the Gblock program and concatenated, excluding the stop codons. Maximum likelihood phylogenies were inferred using IQ-TREE under Edge-linked partition model for 5,000 ultrafast bootstraps and the Shimodaira–Hasegawa–like approximate likelihood-ratio test. ModelFinder (Zhang et al., 2018) was used to select the best-fit partition model (Edge-linked) using the BIC criterion. Best-fit model was defined according to BIC: GTR + F + I + G4. Bayesian inference phylogenies were inferred by MrBayes 3.2.6 using a partition model (2 parallel runs, 5,000,000 generations), in which initial 25% of the sampled data were discarded as burn-in. ITOL was used to present the phylogenetic tree (https://itol.embl.de/).

### Positive Selection

Positive selection was determined using the branch and branch-site models of the codon-based maximum-likelihood (CodeML) method implemented in PAML (Yang, 2007) with the hadal and abyssal species as the foreground. *Hormathia digitata* was removed from this analysis because it was clustered with the abyssal species *Paraphelliactis xishaensis*. The selection pressure was determined based on the overall database of 13 mitochondrial PCGs. The “one-ratio” model (model = 0) assumed that all clades in the phylogenetic tree have equal values of ω. The “free-ratio” model (model = 1) assumed that all clades in the phylogenetic tree have unequal values of ω. The “two-ratio” model (model = 2, the hadal and abyssal species branch was set as the foreground lineages, ω2; all other branches were set as the background lineages, ω1) was applied to the overall database of 13 mitochondrial PCGs. Positively selected sites in the hadal and abyssal species lineages (marked as the foreground lineage) were determined by the branch site models (A and A-null). Bayes empirical Bayes (BEB) analysis was used to calculate the posterior probabilities of the positively selected sites.

### Divergence Time Estimation

The outgroup and species described above were used in divergence time analysis. Following the construction of the phylogenetic tree, MCMCTree of the PAML package (Yang, 2007) was used to estimate the divergence times of the species using a Bayesian method. Two nodes were used in this analysis for time calibration. Actiniaria and Alcyonacea of the Anthozoa class diverged between 512 MYA and 741 MYA (Park et al., 2012; Quattrini et al., 2013), and Antipatharia and Zoantharia diverged between 437 MYA and 600 MYA (Park et al., 2012; Schwentner and Bosch, 2015). In MCMCTree analysis, the independent rates model (clock = 2 in MCMCTree) was used to specify the prior of the rates within internal nodes, which followed a log-normal distribution. The first 200,000 cycles in MCMCTree were discarded as burn-in, and every 50 cycles were sampled to obtain a total of 20,000 samples. Two independent runs were conducted to ensure convergence of the MCMC chains. The program summarized the mean and the 95% confidence intervals (CIs).

## Results and Discussion

### Mitogenome Organization of *Galatheanthemum* sp. MT-2020

**FIGURE 1:**
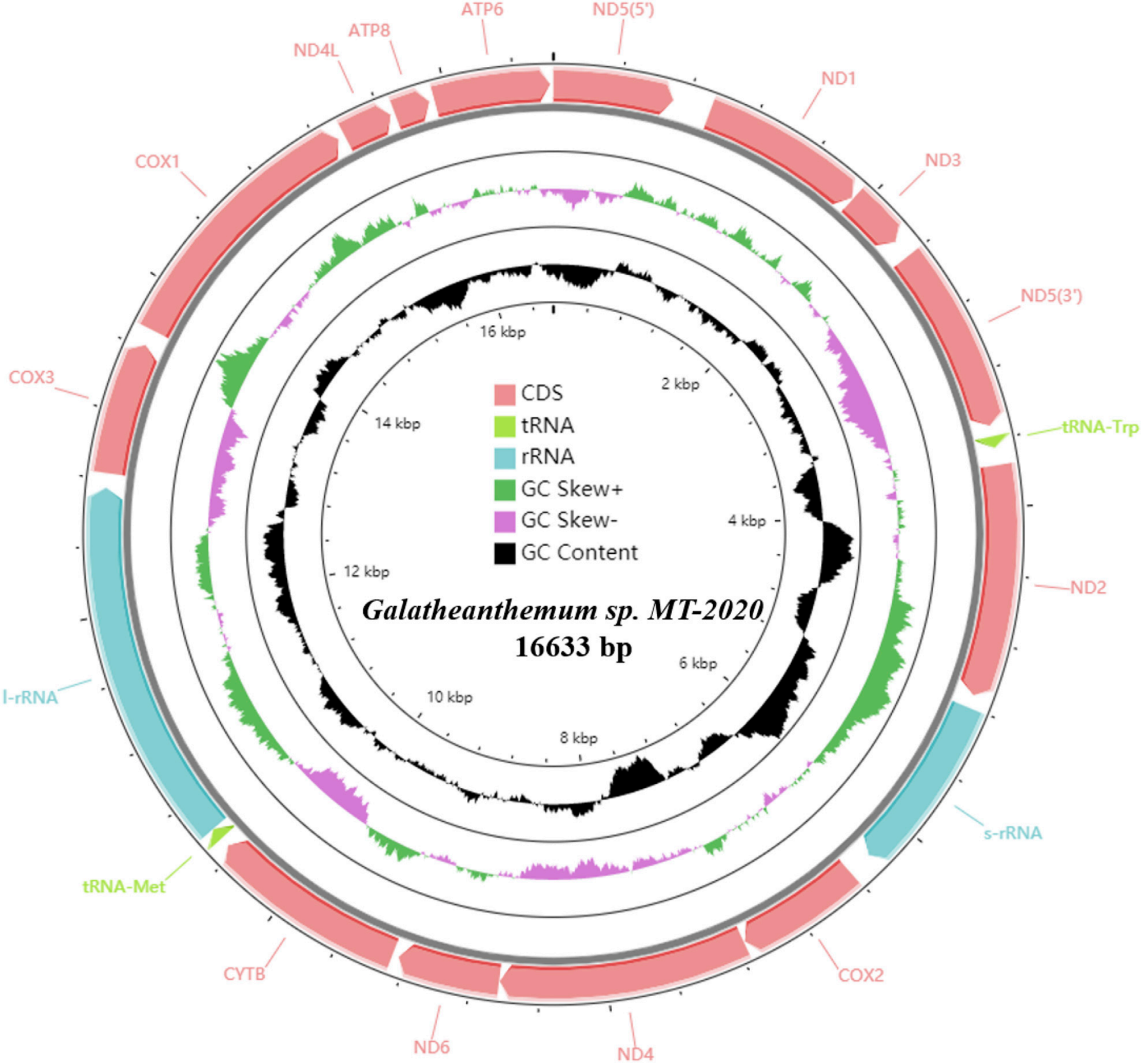
Circular map of the mitogenome of *Galatheanthemum* sp. MT-2020. All of the genes were encoded on the heavy strand.

A total of 619,648,320 clean reads (92,947,248,000 base pairs [bp]) were generated by Illumina HiSeq sequencing. After assembly, the complete mitogenome of *Galatheanthemum* sp. MT-2020 was obtained with a coverage depth of ∼104.5× and identified to be a double strand circular genome of 16,633 bp (Figure 1; Supplementary Table S2). The known sizes of the mitochondrial genomes of sea anemones range from 16,389 to 20,108 bp; *Galatheanthemum* sp. MT-2020 has the second smallest mitochondrial genome sequenced to date containing 13 typical proteins related to the energy pathway (atp6, atp8, cox1-3, cob, nad1-6, and nad4L) and two ribosomal RNA genes (small subunit and large subunit). In addition, in common with most mitochondrial genomes of the subclass Hexacorallia, *Galatheanthemum* sp. MT-2020 contained only two of a total of 22 transfer RNA genes: tRNA-Trp (tryptophan) and tRNA-Met (methionine). All genes were encoded on the heavy (H) strand in the same transcriptional direction, and no overlap was detected in the entire genome; this result was consistent with the features of other reported mitochondrial genomes of sea anemones (Zhang et al., 2017). However, 1,112 bp intergenic nucleotides (IGN) were dispersed among 17 intergenic regions; the longest IGN region was located between ND5 (5’) and ND1, with a length of 223 bp. The special CR motif of “G(A)n T” present in *S. gregaria*, *C. parallelus* and sea anemones *Bolocera* sp. was also observed within the 147 bp intergenic sequence between s-rRNA and cox2; hence, we speculated that this sequence may be a CR-like sequence (Supplementary Figure S2). The gene order of *Galatheanthemum* sp. MT-2020 (ND5, ND1, ND3, tRNA-Trp, ND2, s-rRNA, cox2, ND4, ND6, cytb, tRNA-Met, l-rRNA, cox3, cox1, ND4L, atp8, and atp6) was identical to that of the majority of the species of the order Actiniaria (Zhang et al., 2017; Zhang and Zhu, 2017).

**FIGURE 2:**
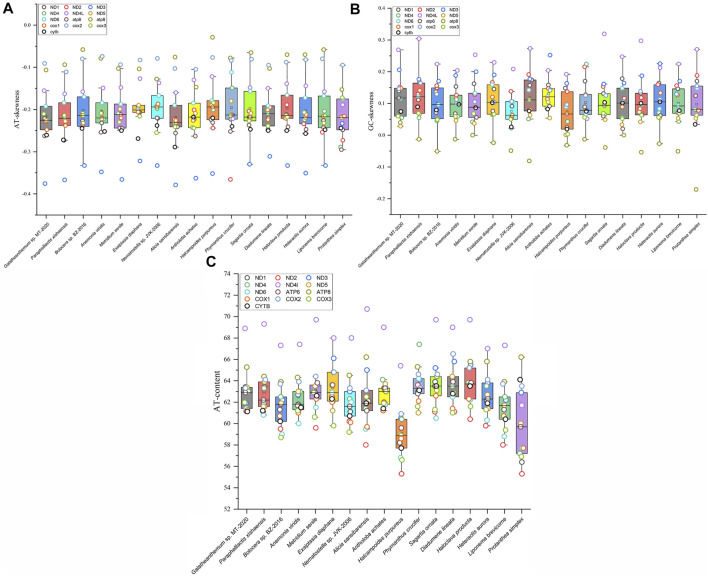
Mitochondrial nucleotide composition of Actiniaria from different families. Box plots showed **(A)** the AT content, **(B)** GC skewness, and **(C)** AT skewness of 13 PCGs in mitochondrial genomes from 16 sea anemone species. PCG was represented by small circle in different colors.

The AT skew and GC skew values of the mitochondrial genomes from 16 families were calculated. The results showed that the AT skew value for Actiniaria was negative in PCGs and in the whole mitogenomes but positive in rRNA. The GC skew value was positive in rRNA, PCGs and the whole mitogenomes. The AT skew values ranged from -0.146 to -0.098 in Actiniaria, and the mitogenome of *Galatheanthemum* sp. MT-2020 contained low counts of A, favouring T, which corresponded to the lowest skew value. The GC skew values of Actiniaria varied from 0.122 to 0.048, and the mitogenome of *Galatheanthemum* sp. MT-2020 contained low counts of C, favouring G (GC skew = 0.121) (Supplementary Table S3). However, this trend was different in the case of PCGs and rRNAs (Supplementary Table S3). Further exploration of the skew values of 13 PCGs of 16 mitogenomes indicated that only *Antholoba achates* and *Galatheanthemum* sp. MT-2020 had a positive GC skew value of the atp8 gene, and the corresponding values of other mitogenomes were negative (Figure 2). The nucleotide skew may represent a balance between selective pressure and mutation during replication (Nikolaou and Almirantis, 2005). Given that *Galatheanthemum* sp. MT-2020 was distributed in the hadal trench, the unusual values of genome-wide AT skew and GC skew of atp8 may be associated with stresses unique to the hadal environment.

### Protein-Coding Genes

**FIGURE 3:**
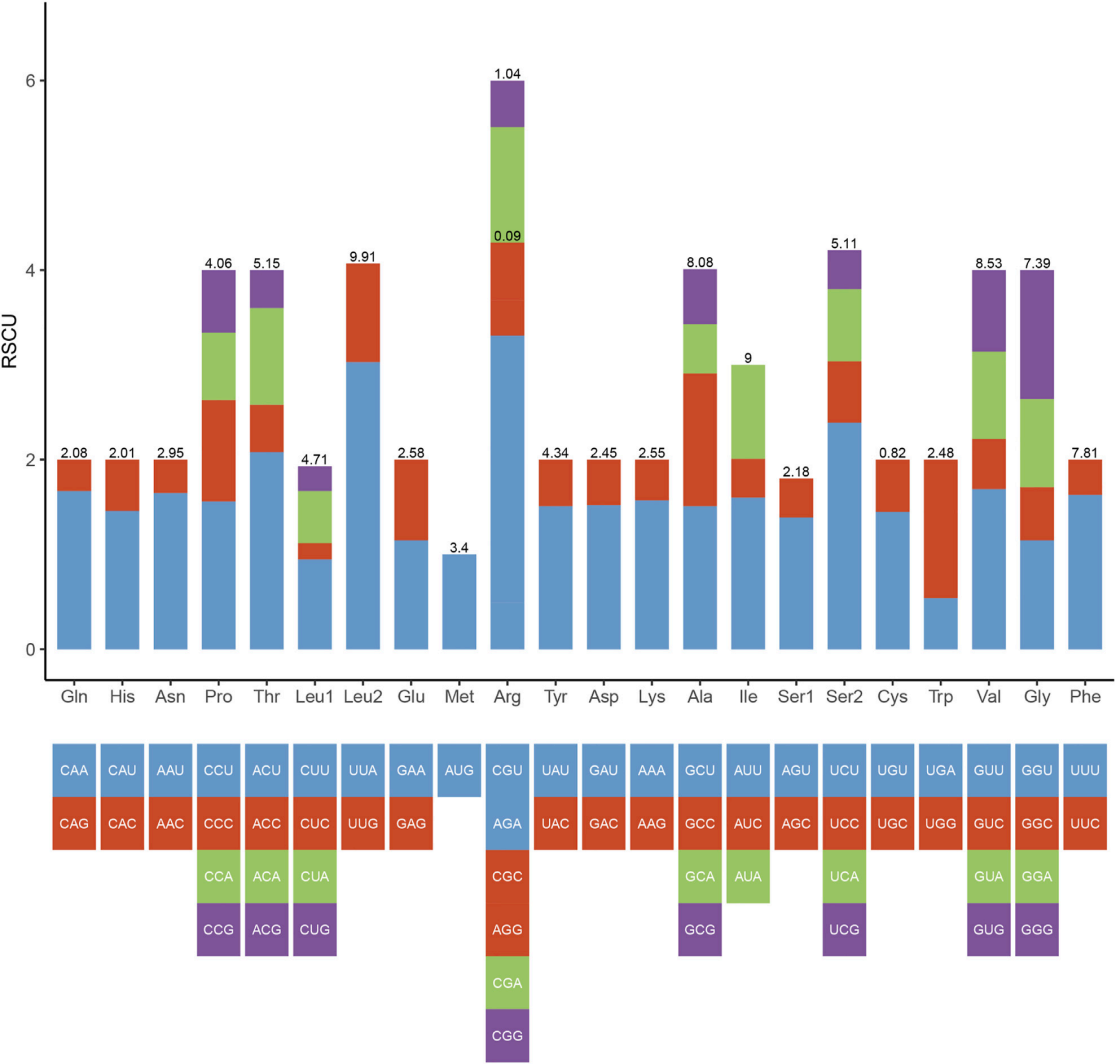
Codon number and relative synonymous codon usage (RSCU) in *Galatheanthemum* sp. MT-2020 mitochondrial PCGs.

In the mitogenome of *Galatheanthemum* sp. MT-2020, the start and stop codons of 13 PCGs were ATG and TAA, respectively, with the exception of ND3, where the stop codon was TAG. This set of the start and stop codons is common in the metazoan mitogenomes (Wolstenholme, 1992). The total length of the PCG sequences in *Galatheanthemum* sp. MT-2020 was 12,105 bp, and the A + T content was 62.39% (Supplementary Table S3). The frequencies of amino acids and relative synonymous codon usage (RSCU) values in the PCGs of *Galatheanthemum* sp. MT-2020 are summarized in Figure 3. The total number of the codons (except stop codons) in the mitogenome of *Galatheanthemum* sp. MT-2020 was 4,035. In the case of PCGs, leucine (14.62%) and arginine (1.13%) were the most and the least used amino acids, respectively. The five most commonly used synonymous codons were TTA (Leu), AGA (Arg), TCT (Ser), ACT (Thr) and GTT (Val) (Figure 3). The usage ratio of amino acids was compared between the members of the abyssal group, including *Galatheanthemum* sp. MT-2020 and *Paraphelliactis xishaensis*, and the members of the group living at the depths of less than 3,000 m. The results showed that the usage ratio of valine in the abyssal group was 8.485 ± 0.04%, which was significantly higher than that in the group living at a depth above 3,000 m. There were no significant differences between the two groups in the case of 19 other amino acids (Supplementary Table S4). Orthologous proteins were compared in the two systems, including nonbarophile (*Pyrococcus furiosus*) and barophile (*Pyrococcus abyssi*) organisms, and the results indicated that the two features of amino acids (polarity and molecular weight) contributed to barophily of these organisms. The pattern of asymmetries in the amino acid substitution process identifies the amino acids arginine, serine, glycine, valine and aspartic acid as those having the most barophilic behaviour. Valine is one of the five amino acids with the strongest barophilic properties (Di Giulio, 2005). In addition, the residues of branched chain amino acids (valine, leucine and isoleucine) often form large hydrophobic clusters, which become the hydrophobic cores of the proteins and enable globular proteins to maintain their stability in a high energy state (Kathuria et al., 2016). Among the branched-chain amino acids, Valine is one of the branched-chain amino acids with the most barophilic behavior, while leucine and isoleucine do not. It is suggested that the branched-chain is neither sufficient nor necessary for an amino acid to have a barophilic behavior, although all of them could form large hydrophobic clusters. Other factors are involved, such as polarity and molecular weight. The 13 mitochondrial PCGs were transmembrane proteins embedded in the hydrophobic lipid chains of the membrane, and an increase in the content of nonpolar amino acids may facilitate a reduction in damage to the membrane caused by pressure (Li et al., 2019). Therefore, increasing the proportion of valine in the proteins may be a means for organism adaptation to high hydrostatic pressure of an extreme environment.

### Ribosomal and Transfer RNA Genes

**FIGURE 4:**
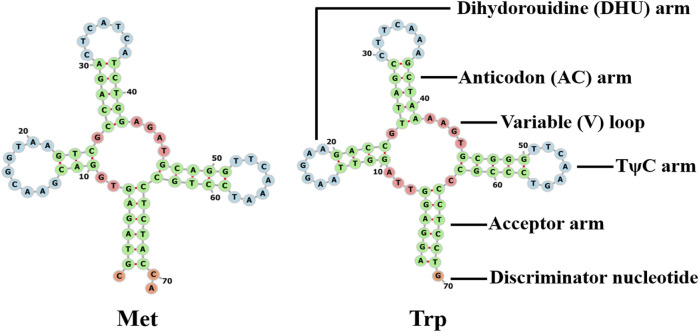
Cloverleaf structure of the two inferred tRNAs in the mitogenomes of *Galatheanthemum* sp. MT-2020 mitogenomes.

The s-rrna and l-rrna genes of *Galatheanthemum* sp. MT-2020 were 1,055 bp (AT% = 54.9) and 2,162 bp (AT% = 58.9) in length, respectively. The s-rrna gene was located between ND2 and cox2, and the l-rrna gene was located between tRNA-Met and cox3. The mitochondrial genome of a typical sea anemone contains only two tRNA genes. Other needed tRNAs are encoded by nuclear DNA and are imported into the mitochondria to function (Beagley and Wolstenholme, 2013). Two tRNAs (tRNA-Trp and tRNA-Met) were detected in *Galatheanthemum* sp. MT-2020 (Figure 4). The secondary structures of these two tRNAs corresponded to a typical clover leaf, and the anticodon was similar to that in most known mitogenomes of sea anemones (Zhang et al., 2017). A mismatched base pair (C-C) was detected in the amino acid acceptor arm of tRNA-Met, and a similar phenomenon has been detected in *Bolocera* sp. where the mismatch is C-A (Zhang et al., 2017). The tRNA-Trp DHU stems also had a mismatch (G-T). A number of the studies have shown that these base mismatches appear to be prevalent in the mitochondrial genomes of many organisms and can be corrected by posttranscriptional RNA editing (Lavrov et al., 2000).

### Nucleotide Diversity

**FIGURE 5:**
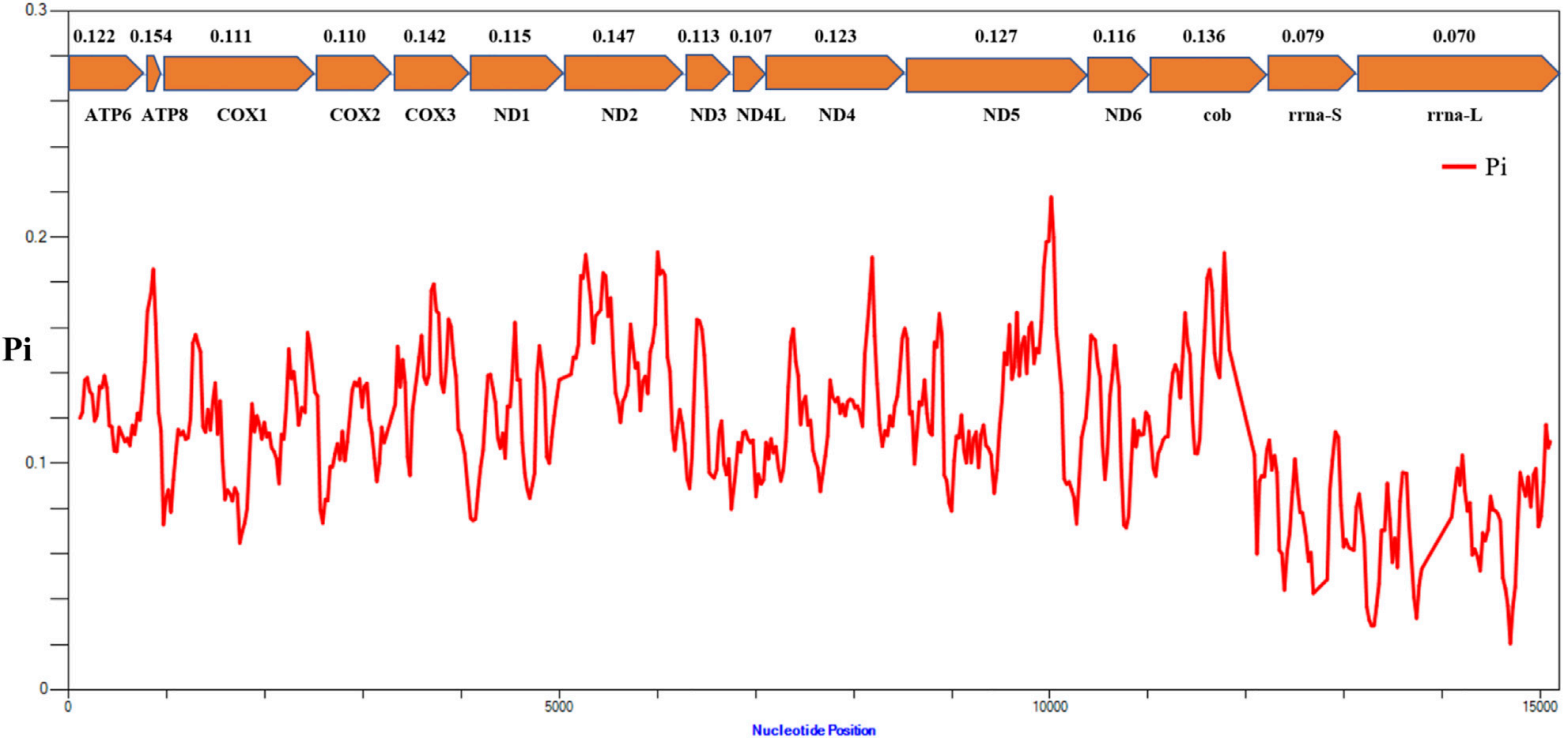
Nucleotide diversity (Pi) of 13 PCGs and two rRNA genes between 16 families of the order Actiniaria. The red line shows the value of Pi in a sliding window analysis. The Pi value of each gene is shown under the gene name.

The results of nucleotide diversity analysis indicated that the values were considerably different between different genes in the mitochondrial genomes of representative species from 16 families of Actiniaria (Figure 5). Among the 13 PCGs, atp8 (Pi = 0.154) presented the highest polymorphism, followed by ND2 (Pi = 0.147) and COX3 (Pi = 0.142), indicating high variability of these genes within the PCGs of the order Actiniaria. ND4L (Pi = 0.107) had the lowest polymorphism, followed by COX2 (Pi = 0.110) and COX1 (Pi = 0.111), which are the conserved PCGs. Both rRNA genes showed very low polymorphism compared to the PCGs, with the nucleotide diversity values of 0.079 for s-rrna and 0.070 for l-rrna.

### Positive Selection Analysis

Mitochondria are the main sites of aerobic respiration in the cells and are crucial for energy metabolism. When the oxygen level in the environment inhabited by an organism decreases or when an organism is subjected to prolonged extreme conditions in which the cells requires massive amounts of energy to counter the challenges posed by external environment, the mitochondria can experience episodic positive selection events, and even purifying selection is the predominant force of evolution of the mitogenomes (Shen et al., 2010). Extreme environments may influence molecular evolution of the organisms. We used the PAML package Codeml to estimate the nonsynonymous/synonymous substitution (dN/dS) ratios.

In branch model analysis, the ω (dN/dS) ratio calculated according to the one-ratio model (M0) was 0.08316 for 13 mitochondrial PCGs of the order Actiniaria, indicating that these genes underwent constrained selection pressure to maintain their functions. Comparison of the LRT values between the one-ratio (M0) and free-ratio models (M1) indicated that the free-ratio model was able to better describe the data than the single-ratio model (Supplementary Table S5), implying that the ω ratios were different in each lineage. In the phylogenetic tree, *Galatheanthemum* sp. MT-2020 and *Paraphelliactis xishaensis* were clustered together; thus, we set this branch as the foreground branch and used other species occurring at the depths below 3,000 m as the background branches. The two-ratio model (M2) was also able to fit the data better than the one-ratio model. The ω ratio of the background branch (ω0 = 0.08422) was 2.45-fold higher than that of the foreground branch (ω1 = 0.03431). The hadal and abyssal species exhibited slower evolutionary rates than the species occurring above 3,000 m, indicating that these species may have experienced nonsynonymous mutations harmful to their survival and that their PCGs had been under strong purifying selection. These results suggested that this slow evolutionary rate may be related to relatively stable environmental conditions in the hadal and abyssal zones. This phenomenon has been also detected in other deep-sea species of the South China deep-sea giant isopods (Shen et al., 2017) and amphipods (Li et al., 2019).

The appearance of a few positively selected sites in the genes tends to occur in a relatively short evolutionary time. A few positively selected sites can be swamped by continued and abundant occurrence of purifying selection sites (Zhang et al., 2005). Thus, when we used the branch-site models to detect possible positive selection sites in the hadal and abyssal species, only one residue was located in the subunit four of NADH dehydrogenase ND4 (328 A), with high Bayes empirical Bayes (BEB) values (>95%) (Supplementary Table S5). In the mitochondria, the electrons are transferred from NADH to O_2_ through a chain of three large enzyme complexes. These complexes include the NADH dehydrogenase complex, which is the first and largest enzyme complex of the respiratory chain, functioning as a proton pump and being the major source of reactive oxygen species in the mitochondria and a significant contributor to cellular oxidative stress (Kussmaul and Hirst, 2006; Yang et al., 2019). In the hadal and abyssal zones, the species need to adapt their energy metabolism to extreme conditions so that the changes in the ND4 site may influence the efficiency of alterations of the NADH dehydrogenase complex. Adaptive evolution of the NADH dehydrogenase complex has been identified in the mitochondrial genome of mammals (Xu et al., 2007; Da Fonseca et al., 2008; Ning et al., 2010; Yu et al., 2011) and Tibetan mudskippers (Wang et al., 2016).

### Phylogenetic Relationships and Gene Arrangements

**FIGURE 6:**
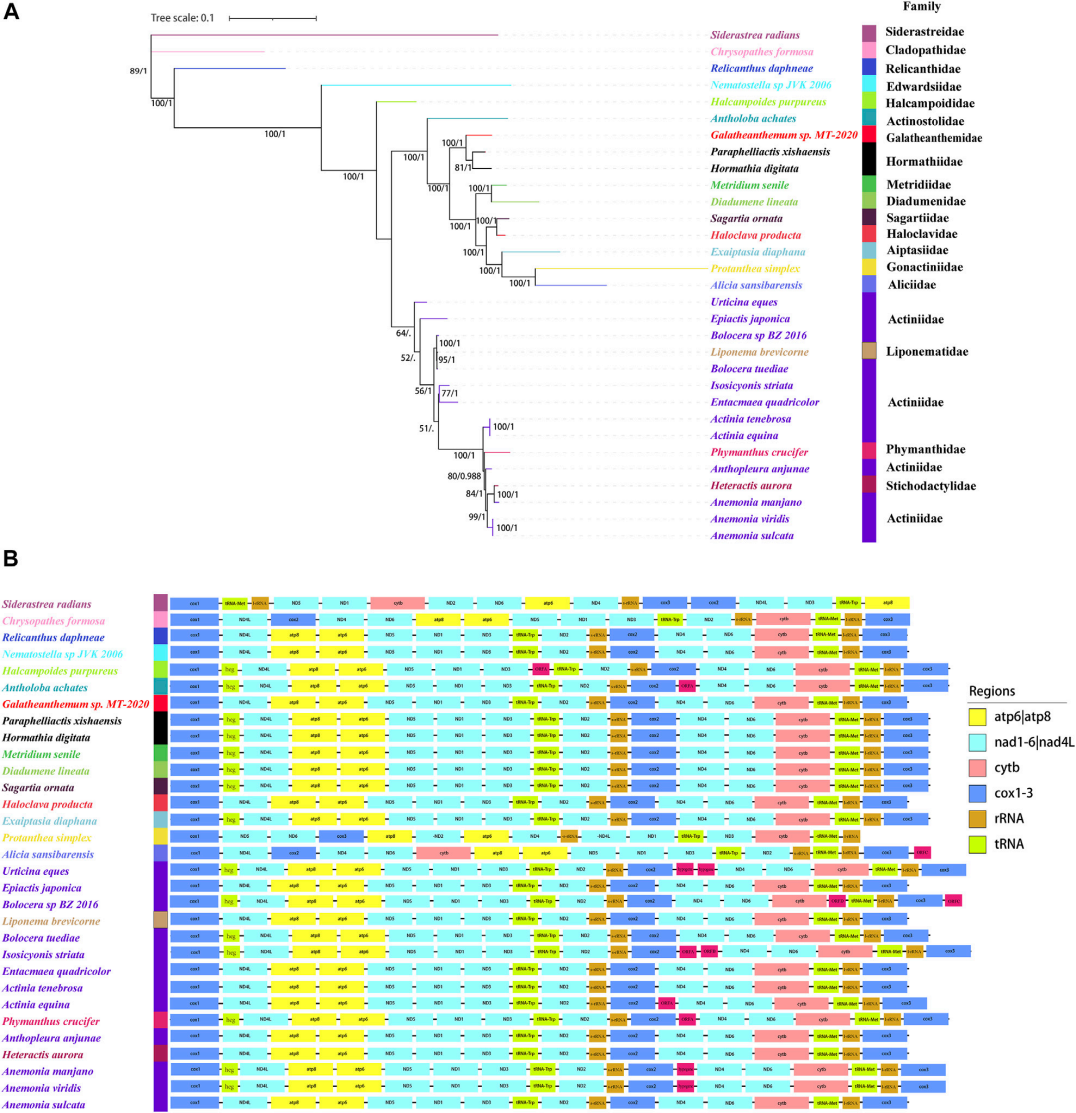
The phylogenetic relationships and mitochondrial gene orders **(A)** The tree was constructed using 28 sea anemones based on the concatenated nucleotide sequences of 13 PCGs by Bayesian and maximum likelihood and showed by ML. Numbers on branches were Bootstrap values (left) and Bayesian posterior probabilities (right). The species examined in this study was labeled with star. **(B)** The gene orders of 28 sea anemones in mitochondrial were mapped, where genes encoded on the light strand are prefixed by minus sign. Different colors indicated different gene groups.

Two phylogenetic trees were constructed in this study. The one constructed with three genes (12S, 16S, and cox3) was showed in Supplementary Figure S1, which showed that *Galatheanthemum* sp. MT-2020 was clustered with Galatheanthemidae species. Another one with the nucleotide sequences of the mitochondrial 13 protein-coding genes was showed in Figure 6A. ML and Bayesian inference (BI) trees displayed a similar overall topology, with certain differences at some branches (Supplementary Figure S3). The ML phylogenetic tree showed that all anemones were clustered in the Clade I and Clade II, except for *Nematostella* sp. JVK-2006 and *Halcampoides purpureus*, which clustered independently. Both *Galatheanthemum* sp. MT-2020 and *Paraphelliactis xishaensis* were present in the Clade I. The relationship (hadal *Galatheanthemum* sp. MT-2020 + (abyssal *Paraphelliactis xishaensis* + cold-water and shallow water *Hormathia digitata*)) had a high nodal support (bootstrap values >80 and posterior possibilities = 1). Furthermore, except for *Antholoba achates*, the species were grouped together and formed a sister clade to the rest of the species of the Clade I, which was composed of three main lineages: 1) Metridiidae and Diadumenidae; 2) Sagartiidae and Haloclavidae; and 3) Aiptasiidae, Gonactiniidae and Aliciidae (Figure 6A). These data indicated that the hadal species *Galatheanthemum* sp. MT-2020 and abyssal species *P. xishaensis* could have originated from the same shallow water ancestor.

The order of 13 PCGs was mostly the same in the present study, and all genes were coded by the H-strand and transcribed in the same direction, except *Protanthea sim*plex and *Alicia sansibarensis,* whose arrangements of 13 PCGs were different (Figure 6B). The order of the genes in the mitogenome of *Protanthea simplex* is heavily scrambled, and the genes are encoded by two DNA strands (Dubin et al., 2019). *Protanthea simplex* and *Alicia sansibarensis* were grouped together in the phylogenetic tree, indicating that these species are the most closely related and could have diverged from the same ancestor. In addition to 13 standard genes encoding energy pathway-related proteins, the mitogenomes of some Actiniaria species had an inserted *heg* gene, one or two group I introns, and some open reading frames (ORFs) with unknown functions. Group I introns are genetic insertion elements, are extremely rare in the metazoans and have been identified only in the mitogenomes of hexacorals and some sponges (Boore, 1999). Some group I introns carry homing endonuclease genes (*heg*) that promote genetic mobility at the DNA level. Homing endonuclease is a sequence-specific DNase (Hafez and Hausner, 2012) that cleaves the intron-lacking alleles in the host genes to spread the introns through homing (Johansen et al., 2021). Open reading frames with unknown functions were detected in Actiniaria of the families Halcampoididae, Actinostolidae, Aliciidae, Actiniidae and Phymanthidae. These noncanonical ORFs are considered to encode the proteins (Flot and Tillier, 2007) and were suggested to be the functional elements with gained, lost or truncated patterns; these properties make these ORFs consistent with the transposon-like elements (Emblem et al., 2014) potentially similar to other elements, such as enhancers or promoters, that regulate the expression of the adjacent genes (Winckler et al., 2005). The mitochondrial genome of *Galatheanthemum* sp. MT-2020 contained only one group I intron in ND5 and did not contain a *heg* gene or noncanonical ORFs.

### Divergence Time

**FIGURE 7:**
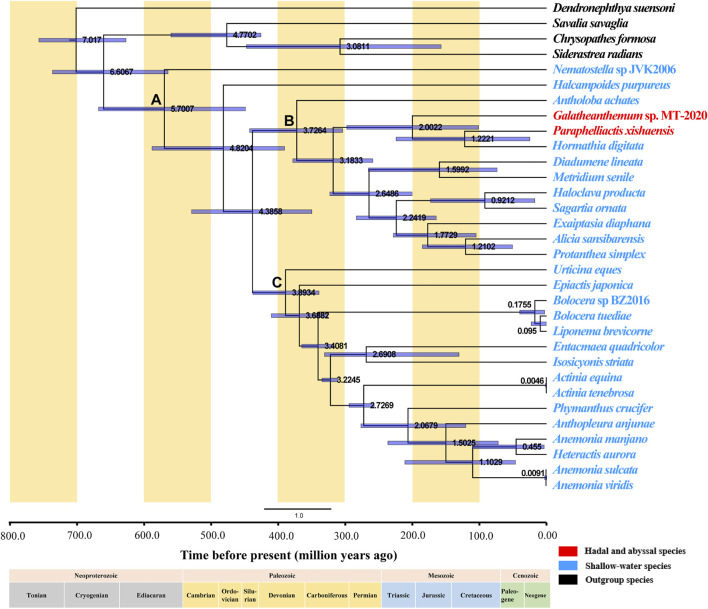
Divergence time of Actiniaria estimated by the Bayesian relaxed-molecular clock method. Node bars indicate 95% credible intervals of the estimated divergence time. The hadal and abyssal species are marked in red, the shallow-water species are marked in blue, and the outgroup species are marked in black. The positions of important nodes are marked with **(A–C)**.

A dendrogram with 95% confidence intervals (CIs) was constructed using Bayesian analysis to estimate the divergence times (Figure 7). Actiniaria was shown to originate in the Cambrian 570.07 million years ago (Mya) (95% highest posterior density interval [HPD]: 449.13–668.13) (Node A). The phylogenies used in previous divergence-dating analyses were generated based on the 50 and 75% concatenated data matrices for 1,729 UCEs (ultraconserved elements) and on exon loci sequenced using a target-enrichment approach; the results of the study placed Actiniaria in the Cambrian with an estimated age of 513 Ma, which is similar to the finding of the present study (McFadden et al., 2021). Furthermore, putative actiniarian fossils have been identified in the Lower Cambrian (Han et al., 2010). The two sister clades, Clade I and Clade II, had formed in the Devonian, and the most recent common ancestor (MRCA) of these two branches has been dated back to 372.64 Mya (95% highest posterior density interval [HPD]: 304.22–442.91) (Node B) and 389.34 (Mya) (95% highest posterior density interval [HPD]: 339.39–438.00) (Node C), respectively. *Galatheanthemum* sp. MT-2020 and *Paraphelliactis xishaensis* were both in Clade I. The MRCA of these two species was in the Early Jurassic 200.22 Mya (95% highest posterior density interval [HPD]: 101.49–298.00). *Paraphelliactis xishaensis* diverged from the shallow marine species *Hormathia digitata* in the Late Jurassic at 122.21 Mya. There is a general consensus that modern deep-sea benthic invertebrate fauna originated in shallow waters after ancient deep-sea animals were eliminated by mass extinction events during the Jurassic and Cretaceous anoxic periods (RexMA, 2010). Other studies have suggested that habitat heterogeneity and intense competition in the upper continental slope and shelf seas could have led to population fractions and evolution of new species, some of which could have tended to colonize the deep sea (Bowen et al., 2013). A comparison with the coeval shelf faunas revealed that a significantly higher number of the families/superfamilies were present in the deep sea than that in the shelf sea since the Early Jurassic, suggesting that deep-sea biota may be more resistant to extinction than shallow-water biota (Thuy et al., 2014) or that the deep-sea environment is more stable and better suited for the survival of the species that are resistant to high pressures. Overall, these findings suggest that *Galatheanthemum* sp. MT-2020 evolved from a shallow marine species and diverged in the Early Jurassic because its characteristics had been better suited for the colonization in the deep sea and it was able to tolerate a greater hydrostatic pressure.

## Data Availability

The datasets presented in this study can be found in online repositories. The names of the repository/repositories and accession number(s) can be found in the article/Supplementary Material.

## References

[B1] Ammons A. W. Daly M. (2008). Distribution, Habitat Use and Ecology of Deepwater Anemones (Actiniaria) in the Gulf of Mexico. Deep Sea Res. Part II Top. Stud. Oceanogr. 55 (24-26), 2657–2666. 10.1016/j.dsr2.2008.07.015

[B2] Beagley C. T. Okada N. A. Wolstenholme D. R. (1996). Two Mitochondrial Group I Introns in a Metazoan, the Sea Anemone Metridium Senile: One Intron Contains Genes for Subunits 1 and 3 of NADH Dehydrogenase. Proc. Natl. Acad. Sci. U.S.A. 93 (11), 5619–5623. 10.1073/pnas.93.11.5619 8643626 PMC39297

[B3] Beagley C. T. Wolstenholme D. R. (2013). Characterization and Localization of Mitochondrial DNA-Encoded tRNAs and Nuclear DNA-Encoded tRNAs in the Sea Anemone Metridium Senile. Curr. Genet. 59 (3), 139–152. 10.1007/s00294-013-0395-9 23801360

[B4] Boore J. L. (1999). Animal Mitochondrial Genomes. Nucleic acids Res. 27 (8), 1767–1780. 10.1093/nar/27.8.1767 10101183 PMC148383

[B5] Bowen B. W. Rocha L. A. Toonen R. J. Karl S. A. (2013). The Origins of Tropical Marine Biodiversity. Trends Ecol. Evol. 28 (6), 359–366. 10.1016/j.tree.2013.01.018 23453048

[B6] Cairns S. D. Bayer F. M. Fautin D. G. (2007). Galatheanthemum Profundale (Anthozoa: Actiniaria) in the Western Atlantic. Bull. Mar. Sci. 80 (1), 191–200.

[B7] Chi S. I. Urbarova I. Johansen S. D. (2018). Expression of Homing Endonuclease Gene and Insertion-like Element in Sea Anemone Mitochondrial Genomes: Lesson Learned from Anemonia Viridis. Gene 652, 78–86. 10.1016/j.gene.2018.01.067 29366757

[B8] Da Fonseca R. R. Johnson W. E. O'Brien S. J. Ramos M. J. Antunes A. (2008). The Adaptive Evolution of the Mammalian Mitochondrial Genome. BMC genomics 9 (1), 119–122. 10.1186/1471-2164-9-119 18318906 PMC2375446

[B9] Di Giulio M. (2005). A Comparison of Proteins from Pyrococcus Furiosus and Pyrococcus Abyssi: Barophily in the Physicochemical Properties of Amino Acids and in the Genetic Code. Gene 346, 1–6. 10.1016/j.gene.2004.10.008 15716096

[B10] Dierckxsens N. Mardulyn P. Smits G. (2017). NOVOPlasty: De Novo Assembly of Organelle Genomes from Whole Genome Data. Nucleic Acids Res. 45 (4), e18. 10.1093/nar/gkw955 28204566 PMC5389512

[B11] Donath A. Jühling F. Al-Arab M. Bernhart S. H. Reinhardt F. Stadler P. F. (2019). Improved Annotation of Protein-Coding Genes Boundaries in Metazoan Mitochondrial Genomes. Nucleic acids Res. 47 (20), 10543–10552. 10.1093/nar/gkz833 31584075 PMC6847864

[B12] Dubin A. Chi S. I. Emblem A. Moum T. Johansen S. D. (2019). Deep-water Sea Anemone with a Two-Chromosome Mitochondrial Genome. Gene 692, 195–200. 10.1016/j.gene.2018.12.074 30641219

[B13] Emblem Å. Okkenhaug S. Weiss E. S. Denver D. R. Karlsen B. O. Moum T. (2014). Sea Anemones Possess Dynamic Mitogenome Structures. Mol. phylogenetics Evol. 75, 184–193. 10.1016/j.ympev.2014.02.016 24613805

[B14] Fautin D. G. Malarky L. Soberon J. (2013). Latitudinal Diversity of Sea Anemones (Cnidaria: Actiniaria). Biol. Bull. 224 (2), 89–98. 10.1086/bblv224n2p89 23677974

[B15] Flot J.-F. Tillier S. (2007). The Mitochondrial Genome of Pocillopora (Cnidaria: Scleractinia) Contains Two Variable Regions: the Putative D-Loop and a Novel ORF of Unknown Function. Gene 401 (1-2), 80–87. 10.1016/j.gene.2007.07.006 17716831

[B16] Gissi C. Iannelli F. Pesole G. (2008). Evolution of the Mitochondrial Genome of Metazoa as Exemplified by Comparison of Congeneric Species. Heredity 101 (4), 301–320. 10.1038/hdy.2008.62 18612321

[B17] Hafez M. Hausner G. (2012). Homing Endonucleases: DNA Scissors on a Mission. Genome 55 (8), 553–569. 10.1139/g2012-049 22891613

[B18] Han J. Kubota S. Uchida H.-o. Stanley G. D. Jr Yao X. Shu D. (2010). Tiny Sea Anemone from the Lower Cambrian of China. PLoS One, 5. e13276. 10.1371/journal.pone.0013276 20967244 PMC2954142

[B19] Jamieson A. J. (2011). Ecology of Deep Oceans: Hadal Trenches. Amsterdam, Netherlands: eLS. 10.1002/9780470015902.a0023606

[B20] Jamieson A. (2015). The Hadal Zone: Life in the Deepest Oceans. Aberdeen, Scotland: Cambridge University Press.

[B21] Johansen S. D. Chi S. I. Dubin A. Jorgensen T. E. (2021). The Mitochondrial Genome of the Sea Anemone Stichodactyla Haddoni Reveals Catalytic Introns, Insertion-like Element, and Unexpected Phylogeny. Life (Basel) 11 (5), 402. 10.3390/life11050402 33924866 PMC8146996

[B22] Johansen S. D. Emblem Å. (2020). “Mitochondrial Group I Introns in Hexacorals Are Regulatory Genetic Elements,” in Advances in the Studies of the Benthic Zone (IntechOpen), 101. 10.5772/intechopen.91465

[B23] Kathuria S. V. Chan Y. H. Nobrega R. P. Özen A. Matthews C. R. (2016). Clusters of Isoleucine, Leucine, and Valine Side Chains Define Cores of Stability in High‐energy States of Globular Proteins: Sequence Determinants of Structure and Stability. Protein Sci. 25 (3), 662–675. 10.1002/pro.2860 26660714 PMC4815418

[B24] Kumar S. Stecher G. Li M. Knyaz C. Tamura K. (2018). MEGA X: Molecular Evolutionary Genetics Analysis across Computing Platforms. Mol. Biol. Evol. 35 (6), 1547. 10.1093/molbev/msy096 29722887 PMC5967553

[B25] Kussmaul L. Hirst J. (2006). The Mechanism of Superoxide Production by NADH: Ubiquinone Oxidoreductase (Complex I) from Bovine Heart Mitochondria. Proc. Natl. Acad. Sci. 103 (20), 7607–7612. 10.1073/pnas.0510977103 16682634 PMC1472492

[B26] Lavrov D. V. Brown W. M. Boore J. L. (2000). A Novel Type of RNA Editing Occurs in the Mitochondrial tRNAs of the Centipede *Lithobius forficatus* . Proc. Natl. Acad. Sci. 97 (25), 13738–13742. 10.1073/pnas.250402997 11095730 PMC17645

[B27] Li J.-y. Zeng C. Yan G.-y. He L.-s. (2019). Characterization of the Mitochondrial Genome of an Ancient Amphipod Halice Sp. MT-2017 (Pardaliscidae) from 10,908 M in the Mariana Trench. Sci. Rep. 9 (1), 1–15. 10.1038/s41598-019-38735-z 30796230 PMC6385184

[B28] Lowe T. M. Eddy S. R. (1997). tRNAscan-SE: a Program for Improved Detection of Transfer RNA Genes in Genomic Sequence. Nucleic acids Res. 25 (5), 955–964. 10.1093/nar/25.5.955 9023104 PMC146525

[B29] McFadden C. S. Quattrini A. M. Brugler M. R. Cowman P. F. Duenas L. F. Kitahara M. V. (2021). Phylogenomics, Origin, and Diversification of Anthozoans (Phylum Cnidaria). Syst. Biol. 70 (4), 635–647. 10.1093/sysbio/syaa103 33507310

[B30] Nikolaou C. Almirantis Y. (2005). A Study on the Correlation of Nucleotide Skews and the Positioning of the Origin of Replication: Different Modes of Replication in Bacterial Species. Nucleic acids Res. 33 (21), 6816–6822. 10.1093/nar/gki988 16321966 PMC1301597

[B31] Ning T. Xiao H. Li J. Hua S. Zhang Y. (2010). Adaptive Evolution of the Mitochondrial ND6 Gene in the Domestic Horse. Genet. Mol. Res. 9 (1), 144–150. 10.4238/vol9-1gmr705 20198570

[B32] Osigus H.-J. Eitel M. Bernt M. Donath A. Schierwater B. (2013). Mitogenomics at the Base of Metazoa. Mol. Phylogenetics Evol. 69 (2), 339–351. 10.1016/j.ympev.2013.07.016 23891951

[B33] Park E. Hwang D.-S. Lee J.-S. Song J.-I. Seo T.-K. Won Y.-J. (2012). Estimation of Divergence Times in Cnidarian Evolution Based on Mitochondrial Protein-Coding Genes and the Fossil Record. Mol. phylogenetics Evol. 62 (1), 329–345. 10.1016/j.ympev.2011.10.008 22040765

[B34] Quattrini A. M. Georgian S. E. Byrnes L. Stevens A. Falco R. Cordes E. E. (2013). Niche Divergence by Deep‐sea Octocorals in the Genus Callogorgia across the Continental Slope of the Gulf of Mexico. Mol. Ecol. 22 (15), 4123–4140. 10.1111/mec.12370 23786376

[B35] Reft A. J. Daly M. (2012). Morphology, Distribution, and Evolution of Apical Structure of Nematocysts in Hexacorallia. J. Morphol. 273 (2), 121–136. 10.1002/jmor.11014 21960117

[B36] RexMA E. (2010). Deep-sea Biodiversity—Pattern and Scale Cambridge. MA: Harvard University Press.

[B37] Rodriguez E. Barbeitos M. S. Brugler M. R. Crowley L. M. Grajales A. Gusmao L. (2014). Hidden Among Sea Anemones: the First Comprehensive Phylogenetic Reconstruction of the Order Actiniaria (Cnidaria, Anthozoa, Hexacorallia) Reveals a Novel Group of Hexacorals. PLoS One 9. e96998. 10.1371/journal.pone.0096998 24806477 PMC4013120

[B38] Schwentner M. Bosch T. C. (2015). Revisiting the Age, Evolutionary History and Species Level Diversity of the Genus Hydra (Cnidaria: Hydrozoa). Mol. Phylogenetics Evol. 91, 41–55. 10.1016/j.ympev.2015.05.013 26014206

[B39] Shen Y.-Y. Liang L. Zhu Z.-H. Zhou W.-P. Irwin D. M. Zhang Y.-P. (2010). Adaptive Evolution of Energy Metabolism Genes and the Origin of Flight in Bats. Proc. Natl. Acad. Sci. 107 (19), 8666–8671. 10.1073/pnas.0912613107 20421465 PMC2889356

[B40] Shen Y. Kou Q. Zhong Z. Li X. He L. He S. (2017). The First Complete Mitogenome of the South China Deep‐sea Giant Isopod Bathynomus sp.(Crustacea: Isopoda: Cirolanidae) Allows Insights into the Early Mitogenomic Evolution of Isopods. Ecol. Evol. 7 (6), 1869–1881. 10.1002/ece3.2737 28331594 PMC5355201

[B41] Thuy B. Kiel S. Dulai A. Gale A. S. Kroh A. Lord A. R. (2014). First Glimpse into Lower Jurassic Deep-Sea Biodiversity: *In Situ* Diversification and Resilience against Extinction. Proc. R. Soc. B Biol. Sci. 281 (1786), 20132624. 10.1098/rspb.2013.2624 PMC4046392 24850917

[B42] Wang Y. Shen Y. Feng C. Zhao K. Song Z. Zhang Y. (2016). Mitogenomic Perspectives on the Origin of Tibetan Loaches and Their Adaptation to High Altitude. Sci. Rep. 6 (1), 1–10. 10.1038/srep29690 27417983 PMC4945904

[B43] Winckler T. Szafranski K. Glöckner G. (2005). Transfer RNA Gene-Targeted Integration: an Adaptation of Retrotransposable Elements to Survive in the Compact *Dictyostelium discoideum* Genome. Cytogenet. genome Res. 110 (1-4), 288–298. 10.1159/000084961 16093681

[B44] Wolff T. (1970). Thee Concept of the Hadal or Ultra-abyssal Fauna. Deep Sea Res. Oceanogr. Abstr., 17 983–1003. 10.1016/0011-7471(70)90049-5

[B45] Wolff T. (1959). The Hadal Community, an Introduction. Deep Sea Res. 6, 95–124. 10.1016/0146-6313(59)90063-2

[B46] Wolstenholme D. R. (1992). Animal Mitochondrial DNA: Structure and Evolution. Int. Rev. Cytol. 141, 173–216. 10.1016/s0074-7696(08)62066-5 1452431

[B47] Xu S. Luosang J. Hua S. He J. Ciren A. Wang W. (2007). High Altitude Adaptation and Phylogenetic Analysis of Tibetan Horse Based on the Mitochondrial Genome. J. Genet. Genomics 34 (8), 720–729. 10.1016/s1673-8527(07)60081-2 17707216

[B48] Yang M. Gong L. Sui J. Li X. (2019). The Complete Mitochondrial Genome of Calyptogena Marissinica (Heterodonta: Veneroida: Vesicomyidae): Insight into the Deep-Sea Adaptive Evolution of Vesicomyids. PloS one 14. e0217952. 10.1371/journal.pone.0217952 31536521 PMC6752807

[B49] Yang Z. (2007). PAML 4: Phylogenetic Analysis by Maximum Likelihood. Mol. Biol. Evol. 24 (8), 1586–1591. 10.1093/molbev/msm088 17483113

[B50] Yu L. Wang X. Ting N. Zhang Y. (2011). Mitogenomic Analysis of Chinese Snub-Nosed Monkeys: Evidence of Positive Selection in NADH Dehydrogenase Genes in High-Altitude Adaptation. Mitochondrion 11 (3), 497–503. 10.1016/j.mito.2011.01.004 21292038

[B51] Zhang B. Zhang Y. H. Wang X. Zhang H. X. Lin Q. (2017). The Mitochondrial Genome of a Sea Anemone Bolocera Sp. Exhibits Novel Genetic Structures Potentially Involved in Adaptation to the Deep-Sea Environment. Ecol. Evol. 7 (13), 4951–4962. 10.1002/ece3.3067 28690821 PMC5496520

[B52] Zhang D. Gao F. Li W. Jakovlić I. Zou H. Zhang J. (2020). PhyloSuite: an Integrated and Scalable Desktop Platform for Streamlined Molecular Sequence Data Management and Evolutionary Phylogenetics Studies, Mol Ecol Resour 20, 348–355. bioRxiv 489088. Mitochondrial DNA Part B 1011. 10.1111/1755-0998.13096 31599058

[B53] Zhang J. Nielsen R. Yang Z. (2005). Evaluation of an Improved Branch-Site Likelihood Method for Detecting Positive Selection at the Molecular Level. Mol. Biol. Evol. 22 (12), 2472–2479. 10.1093/molbev/msi237 16107592

[B54] Zhang L. Zhu Q. (2017). Complete Mitochondrial Genome of the Sea Anemone, Anthopleura Midori (Actiniaria: Actiniidae). Mitochondrial DNA Part A 28 (3), 335–336. 10.3109/19401736.2015.1122770 27159694

