# Supplementary material for: Comparative Analysis of the Mitochondrial Genome of Galatheanthemum sp. MT-2020 (Actiniaria Galatheanthemidae) From a Depth of 9,462 m at the Mariana Trench
Source: Front Genet. 2022 Jun 8;13:854009. doi: 10.3389/fgene.2022.854009 (PMC9213748; doi:10.3389/fgene.2022.854009)
Supplement: Supplementary file 1 [file DataSheet1.docx]

**
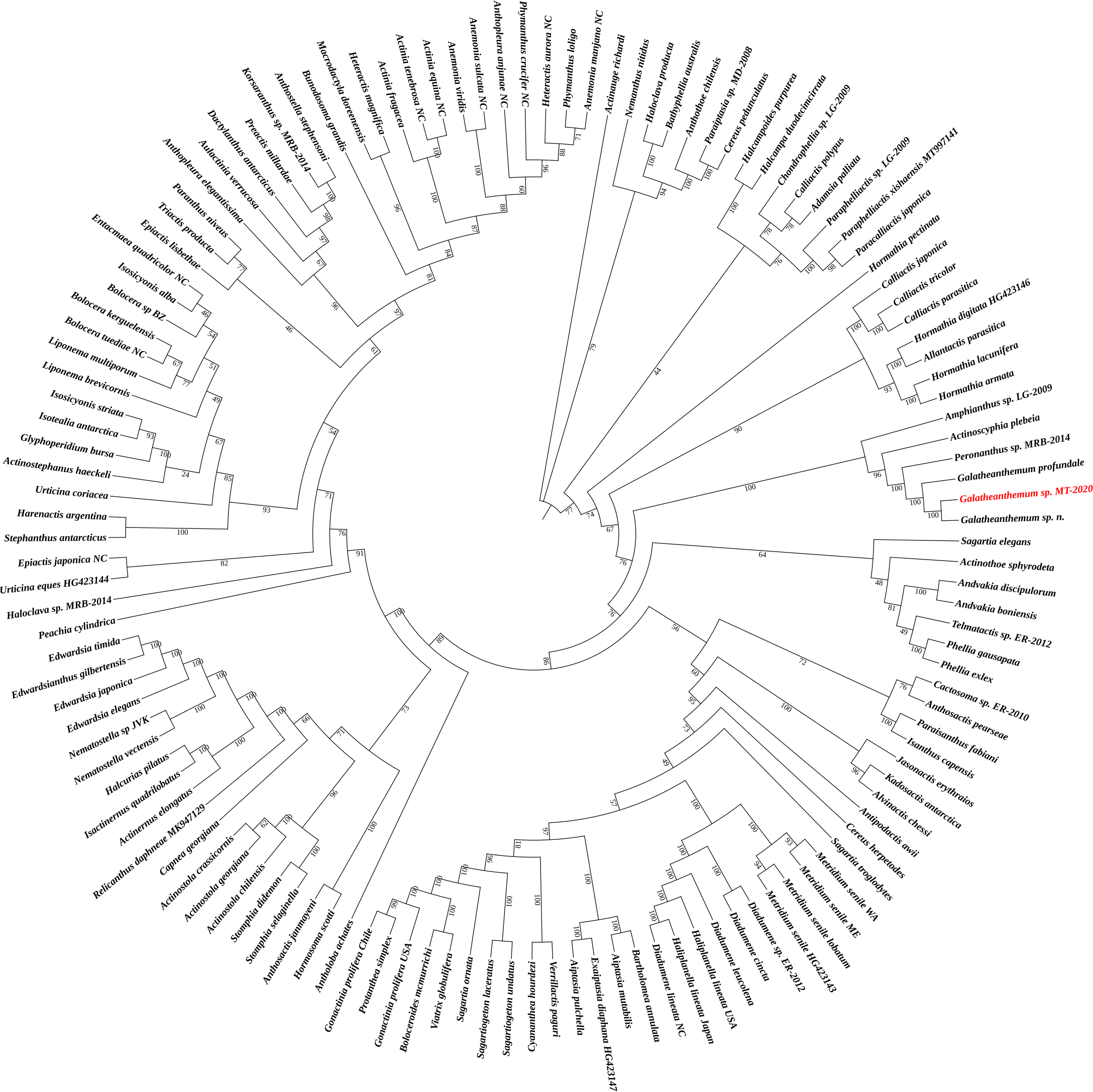
**

**Figure S1.** Phylogenetic tree analysis with concatenated 12S, 16S and cox3 using the Maximum Likelihood approach. The red font indicates the species in this study.


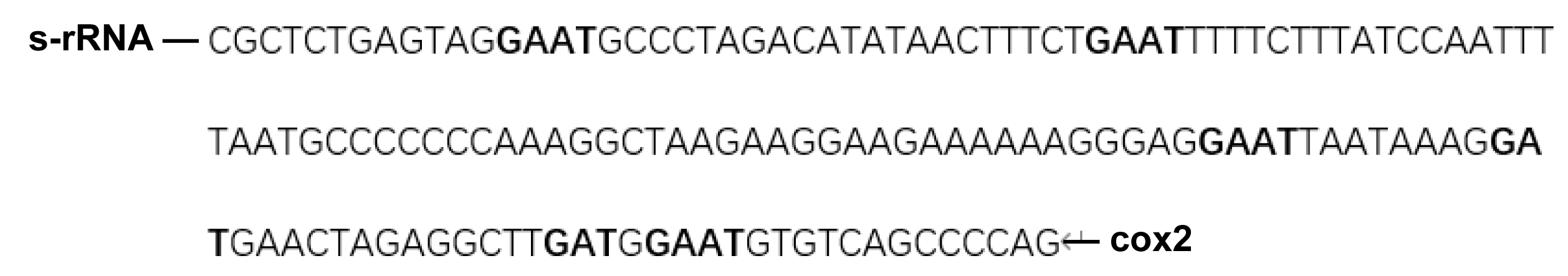


**Figure S2.** The CR-like sequences of *Galatheanthemum* sp. MT-2020. The “G(A)n T” motifs were marked with bold font.


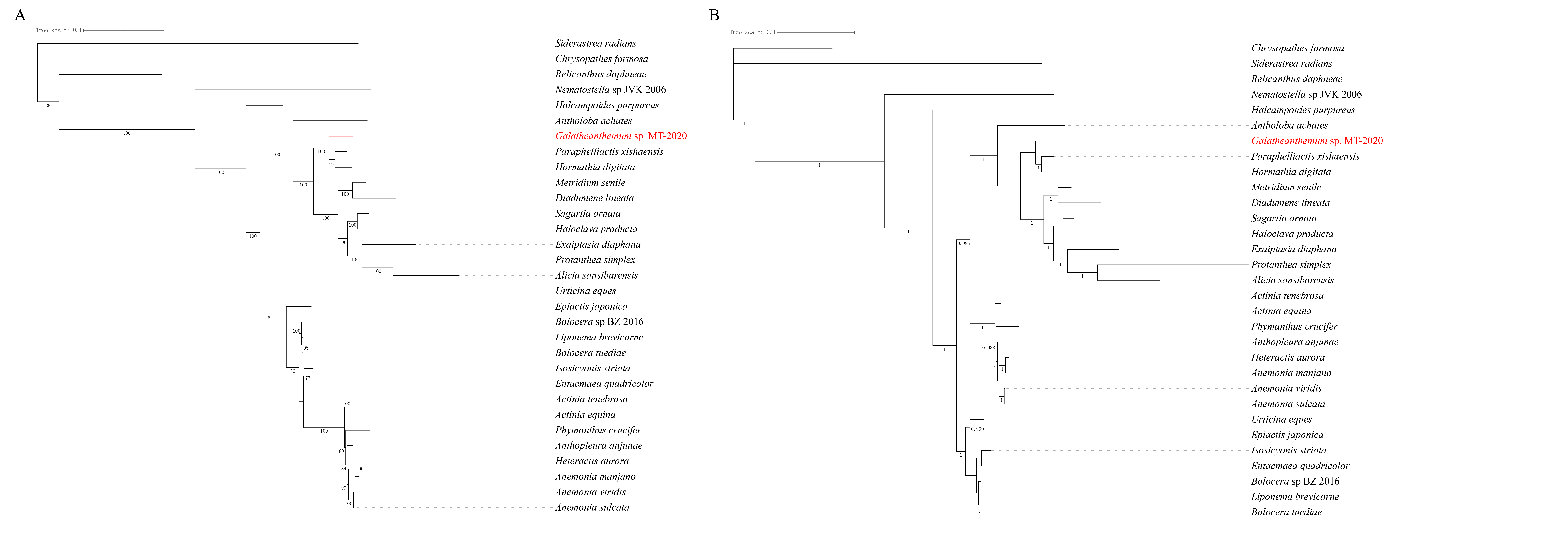


**Figure S3.** Phylogenetic trees showing relationships among 28 sea anemones based on a dataset of 13 PCG sequences. A is the tree of ML analysis, and B is the tree of BI analysis. Numbers next to nodes specify bootstrap percentages from ML analysis and the Bayesian posterior probabilities (BPPs). Only bootstrap percentages greater than 50% are shown. The ML tree and BI tree were generated by IQ-TREE v1.6.8 and MrBayes v3.2.6, respectively.

**Table S1.** Summary of the species with available mitochondrial genome used in this study.

|  | Species | Class | Order | Family | Genus | Accession number |
| --- | --- | --- | --- | --- | --- | --- |
| Ingroup | *Galatheanthemum* sp. MT-2020 | Anthozoa | Actiniaria | Galatheanthemidae | Galatheanthemum | OL912950 |
|  | *Metridium senile* | Anthozoa | Actiniaria | Metridiidae | Metridium | HG423143.1 |
|  | *Urticina eques* | Anthozoa | Actiniaria | Actiniidae | Urticina | HG423144.1 |
|  | *Hormathia digitata* | Anthozoa | Actiniaria | Hormathiidae | Hormathia | HG423146.1 |
|  | *Exaiptasia diaphana* | Anthozoa | Actiniaria | Aiptasiidae | Exaiptasia | HG423147.1 |
|  | *Isosicyonis striata* | Anthozoa | Actiniaria | Actiniidae | Isosicyonis | KR051006.1 |
|  | *Bolocera* sp. BZ-2016 | Anthozoa | Actiniaria | Actiniidae | Bolocera | KU507297.1 |
|  | *Protanthea simplex* | Anthozoa | Actiniaria | Gonactiniidae | Protanthea | MH500774.1 |
|  | *Relicanthus daphneae* | Anthozoa | Actiniaria | Relicanthidae | Relicanthus | MK947129.1 |
|  | *Paraphelliactis xishaensis* | Anthozoa | Actiniaria | Hormathiidae | Paraphelliactis | MT997141.1 |
|  | *Nematostella* sp. JVK-2006 | Anthozoa | Actiniaria | Edwardsiidae | Nematostella | NC_008164.1 |
|  | *Bolocera tuediae* | Anthozoa | Actiniaria | Actiniidae | Bolocera | NC_022470.1 |
|  | *Alicia sansibarensis* | Anthozoa | Actiniaria | Aliciidae | Alicia | NC_027610.1 |
|  | *Antholoba achates* | Anthozoa | Actiniaria | Actinostolidae | Antholoba | NC_027611.1 |
|  | *Halcampoides purpureus* | Anthozoa | Actiniaria | Halcampoididae | Halcampoides | NC_027612.1 |
|  | *Phymanthus crucifer* | Anthozoa | Actiniaria | Phymanthidae | Phymanthus | NC_027614.1 |
|  | *Sagartia ornata* | Anthozoa | Actiniaria | Sagartiidae | Sagartia | NC_027615.1 |
|  | *Anthopleura anjunae* | Anthozoa | Actiniaria | Actiniidae | Anthopleura | NC_030274.1 |
|  | *Anemonia viridis* | Anthozoa | Actiniaria | Actiniidae | Anemonia | NC_037177.1 |
|  | *Anemonia manjano* | Anthozoa | Actiniaria | Actiniidae | Anemonia | NC_037178.1 |
|  | *Actinia equina* | Anthozoa | Actiniaria | Actiniidae | Actinia | NC_039929.1 |
|  | *Actinia tenebrosa* | Anthozoa | Actiniaria | Actiniidae | Actinia | NC_044902.1 |
|  | *Diadumene lineata* | Anthozoa | Actiniaria | Diadumenidae | Diadumene | NC_045515.1 |
|  | *Epiactis japonica* | Anthozoa | Actiniaria | Actiniidae | Epiactis | NC_047217.1 |
|  | *Haloclava producta* | Anthozoa | Actiniaria | Haloclavidae | Haloclava | NC_047218.1 |
|  | *Heteractis aurora* | Anthozoa | Actiniaria | Stichodactylidae | Heteractis | NC_047219.1 |
|  | *Liponema brevicorne* | Anthozoa | Actiniaria | Liponematidae | Liponema | NC_047221.1 |
|  | *Anemonia sulcata* | Anthozoa | Actiniaria | Actiniidae | Anemonia | NC_049065.1 |
|  | *Entacmaea quadricolor* | Anthozoa | Actiniaria | Actiniidae | Entacmaea | NC_049066.1 |
| Outgroup | *Chrysopathes formosa* | Anthozoa | Antipatharia | Cladopathidae | Chrysopathes | DQ304771.1 |
|  | *Siderastrea radians* | Anthozoa | Scleractinia | Siderastreidae | Siderastrea | DQ643838.1 |

**Table S2.** Mitogenome organization of *Galatheanthemum* sp. MT-2020.

| Gene | Position |  | Size | | Intergenic nucleotides | | Codon | |  |
| --- | --- | --- | --- | --- | --- | --- | --- | --- | --- |
|  | From | To | Nucleotides | Amino acid | |  | Start | Stop | Strand |
| ND5 | 1 | 3514 | 1833 | 611 | 223 | | ATG | TAA | H |
| ND1 | 941 | 1924 | 984 | 328 | 6 | | ATG | TAA | H |
| ND3 | 1931 | 2287 | 357 | 119 | 174 | | ATG | TAG | H |
| tRNA-Trp | 3578 | 3647 | 70 |  | 102 | |  |  | H |
| ND2 | 3750 | 5126 | 1377 | 459 | 78 | | ATG | TAA | H |
| s-rRNA | 5205 | 6259 | 1055 |  | 147 | |  |  | H |
| cox2 | 6407 | 7153 | 747 | 249 | 5 | | ATG | TAA | H |
| ND4 | 7159 | 8634 | 1476 | 492 | 4 | | ATG | TAA | H |
| ND6 | 8638 | 9247 | 609 | 203 | 37 | | ATG | TAA | H |
| cytb | 9285 | 10457 | 1173 | 391 | 55 | | ATG | TAA | H |
| tRNA-Met | 10513 | 10585 | 73 |  | 1 | |  |  | H |
| l-rRNA | 10587 | 12748 | 2162 |  | 86 | |  |  | H |
| cox3 | 12835 | 13623 | 789 | 263 | 97 | | ATG | TAA | H |
| cox1 | 13721 | 15313 | 1593 | 531 | 41 | | ATG | TAA | H |
| ND4L | 15355 | 15654 | 300 | 100 | 24 | | ATG | TAA | H |
| atp8 | 15679 | 15894 | 216 | 72 | 30 | | ATG | TAA | H |
| atp6 | 15925 | 16614 | 690 | 230 | 2 | | ATG | TAA | H |
| Overlap: | 0 | gap: | 17 |  | 1112 | |  |  |  |

**Table S3.** Nucleotide composition of the mitochondrial genomes in different sea anemones.

| Species | Length(bp) | A% | T% | G% | C% | A+T% | AT skew | GC skew |
| --- | --- | --- | --- | --- | --- | --- | --- | --- |
| **Whole mitochondrial genome** | | | | | | | | |
| *Galatheanthemum* sp. MT-2020 | 16633 | 26.2 | 35.1 | 21.7 | 17 | 61.3 | -0.146 | 0.121 |
| *Paraphelliactis xishaensis* | 18592 | 26.7 | 34.9 | 21.6 | 16.9 | 61.6 | -0.134 | 0.120 |
| *Bolocera* sp. BZ-2016 | 19463 | 26.4 | 34.1 | 21.9 | 17.6 | 60.5 | -0.127 | 0.108 |
| *Anemonia viridis* | 20108 | 27 | 34.3 | 21.4 | 17.3 | 61.3 | -0.120 | 0.107 |
| *Metridium senile* | 17444 | 27 | 34.9 | 21.2 | 16.9 | 61.9 | -0.129 | 0.112 |
| *Exaiptasia diaphana* | 19791 | 28 | 34.5 | 20.9 | 16.7 | 62.5 | -0.104 | 0.110 |
| *Nematostella* sp. JVK-2006 | 16389 | 26.9 | 34 | 21.3 | 17.8 | 60.9 | -0.117 | 0.090 |
| *Alicia sansibarensis* | 19575 | 26.7 | 34.3 | 21.6 | 17.3 | 61 | -0.126 | 0.110 |
| *Antholoba achates* | 17816 | 26.9 | 35 | 21.3 | 16.7 | 61.9 | -0.130 | 0.122 |
| *Halcampoides purpureus* | 18038 | 25.8 | 32.1 | 22.8 | 19.3 | 57.9 | -0.108 | 0.083 |
| *Phymanthus crucifer* | 19727 | 27.2 | 35.2 | 21 | 16.6 | 62.4 | -0.128 | 0.117 |
| *Sagartia ornata* | 17446 | 27.2 | 35.1 | 20.9 | 16.6 | 62.3 | -0.127 | 0.114 |
| *Diadumene lineata* | 17552 | 27.2 | 35.3 | 20.8 | 16.6 | 62.5 | -0.129 | 0.114 |
| *Haloclava producta* | 17416 | 27.3 | 35.2 | 20.9 | 16.6 | 62.5 | -0.126 | 0.114 |
| *Heteractis aurora* | 19999 | 27.1 | 34.5 | 21.3 | 17.1 | 61.6 | -0.121 | 0.109 |
| *Liponema brevicorne* | 19143 | 26.4 | 33.9 | 22 | 17.7 | 60.3 | -0.123 | 0.109 |
| *Protanthea simplex* | 17134 | 26.1 | 31.8 | 22 | 20 | 57.9 | -0.098 | 0.048 |
| **Protein coding genes** | | | | | | | | |
| *Galatheanthemum* sp. MT-2020 | 12105 | 24.2 | 38.2 | 20.8 | 16.8 | 62.4 | -0.224 | 0.106 |
| *Paraphelliactis xishaensis* | 12114 | 24.2 | 38.3 | 20.8 | 16.7 | 62.5 | -0.226 | 0.109 |
| *Bolocera* sp. BZ-2016 | 12069 | 24 | 37.2 | 21.3 | 17.5 | 61.2 | -0.216 | 0.098 |
| *Anemonia viridis* | 12144 | 24.3 | 37.7 | 20.8 | 17.2 | 62 | -0.216 | 0.095 |
| *Metridium senile* | 11859 | 24.6 | 38 | 20.4 | 17 | 62.6 | -0.214 | 0.091 |
| *Exaiptasia diaphana* | 12270 | 25 | 37.7 | 20.6 | 16.7 | 62.7 | -0.203 | 0.105 |
| *Nematostella* sp. JVK-2006 | 11811 | 24.6 | 36.8 | 20.6 | 18 | 61.4 | -0.199 | 0.067 |
| *Alicia sansibarensis* | 11673 | 23.6 | 37.8 | 21.3 | 17.2 | 61.4 | -0.231 | 0.106 |
| *Antholoba achates* | 12060 | 24.5 | 38.1 | 20.8 | 16.6 | 62.6 | -0.217 | 0.112 |
| *Halcampoides purpureus* | 11793 | 23.5 | 35.1 | 22 | 19.4 | 58.6 | -0.198 | 0.063 |
| *Phymanthus crucifer* | 11678 | 24.8 | 38.1 | 20.5 | 16.6 | 62.9 | -0.211 | 0.105 |
| *Sagartia ornata* | 11859 | 25 | 38.2 | 20.2 | 16.7 | 63.2 | -0.209 | 0.095 |
| *Diadumene lineata* | 11925 | 24.9 | 38.3 | 20 | 16.8 | 63.2 | -0.212 | 0.087 |
| *Haloclava producta* | 11859 | 25 | 38.2 | 20.1 | 16.7 | 63.2 | -0.209 | 0.092 |
| *Heteractis aurora* | 11826 | 24.4 | 37.8 | 20.9 | 17 | 62.2 | -0.215 | 0.103 |
| *Liponema brevicorne* | 11847 | 23.9 | 37.1 | 21.3 | 17.6 | 61 | -0.216 | 0.095 |
| *Protanthea simplex* | 11967 | 22.7 | 36.2 | 22.6 | 18.4 | 58.9 | -0.229 | 0.102 |
| **rRNA** |  |  |  |  |  |  |  |  |
| *Galatheanthemum* sp. MT-2020 | 3217 | 32.3 | 25.3 | 24.9 | 17.5 | 57.6 | 0.122 | 0.175 |
| *Paraphelliactis xishaensis* | 3246 | 32.1 | 25.4 | 25 | 17.5 | 57.5 | 0.117 | 0.176 |
| *Bolocera* sp. BZ-2016 | 3271 | 31.9 | 25.2 | 25.2 | 17.7 | 57.1 | 0.117 | 0.175 |
| *Anemonia viridis* | 3328 | 32.1 | 25.7 | 24.8 | 17.4 | 57.8 | 0.111 | 0.175 |
| *Metridium senile* | 3270 | 32.3 | 25.7 | 24.8 | 17.2 | 58 | 0.114 | 0.181 |
| *Exaiptasia diaphana* | 3244 | 32.7 | 26.6 | 24.4 | 16.3 | 59.3 | 0.103 | 0.199 |
| *Nematostella* sp. JVK-2006 | 2966 | 32.9 | 25.1 | 24.1 | 17.9 | 58 | 0.134 | 0.148 |
| *Alicia sansibarensis* | 3274 | 32.4 | 26.6 | 24.7 | 16.3 | 59 | 0.098 | 0.205 |
| *Antholoba achates* | 3135 | 32.2 | 25.2 | 24.9 | 17.7 | 57.4 | 0.122 | 0.169 |
| *Halcampoides purpureus* | 3272 | 32 | 24.8 | 25.2 | 18 | 56.8 | 0.127 | 0.167 |
| *Phymanthus crucifer* | 3272 | 32.1 | 25.8 | 24.8 | 17.3 | 57.9 | 0.109 | 0.178 |
| *Sagartia ornata* | 3278 | 32.4 | 25.8 | 24.6 | 17.1 | 58.2 | 0.113 | 0.180 |
| *Diadumene lineata* | 3269 | 32.2 | 25.8 | 24.8 | 17.1 | 58 | 0.110 | 0.184 |
| *Haloclava producta* | 2980 | 31.9 | 26.1 | 24.7 | 17.3 | 58 | 0.100 | 0.176 |
| *Heteractis aurora* | 3083 | 31.3 | 26.2 | 25 | 17.4 | 57.5 | 0.089 | 0.179 |
| *Liponema brevicorne* | 3082 | 31.5 | 25.5 | 25.2 | 17.8 | 57 | 0.105 | 0.172 |
| *Protanthea simplex* | 3348 | 31 | 24 | 26.4 | 18.7 | 55 | 0.127 | 0.171 |

**Table S4.** The proportions (%) of amino acid of mitochondrial PCGs from Sea anemones of different families.

**Table S5.** CODEML analyses of selective pressure on mitochondrial genes in the hadal and abyssal lineage.

| **Branch model** |  |  | |  |  |  |  |  |  |
| --- | --- | --- | --- | --- | --- | --- | --- | --- | --- |
| **Trees** | **Model** | **lnL** | **Estimates of parameter** | |  | **Model compared** | **2△lnL** | **LRT p-value** |  |
| ML | Model 1 | -63863.29667 |  | |  | Model 1 versus Model 0 | 447.9767 | 0.00000 |  |
|  | Two ratio | -64080.0728 |  | | ω0=0.08422 ；ω1=0.03431 | Two ratio versus Model 0 | 14.424498 | 0.000145892 |  |
|  | Model 0 | -64087.28504 |  | | ω(dN/dS) = 0.08316 |  |  |  |  |
| **Branch site model** |  |  |  | |  |  |  |  |  |
| **gene** | **Model** | **lnL** | **Estimates of parameter** | |  | **Model compared** | **2△lnL** | **LRT p-value** | **Positive site** |
| ND4 | Model A | -7749.849649 | proportion | | P0=0.9235, P1=0.07287 | Model A versus Model A null | 2.624888 | 0.1051999 | 328 A 0.966* |
|  |  |  |  | | P2a=0.00336, P2b=0.00027 |  |  |  |  |
|  |  |  | background ω | | ω0=0.04649, ω1=1.00000 |  |  |  |  |
|  |  |  |  | | ω2a=0.04649, ω2b=1.00000 |  |  |  |  |
|  |  |  | foreground ω | | ω0=0.04649, ω1=1.00000 |  |  |  |  |
|  |  |  |  | | ω2a=13.34569, ω2b=13.34569 |  |  |  |  |
|  | Model A null | -7751.162093 | proportion | | P0=0.90783, P1=0.07194 |  |  |  |  |
|  |  |  |  | | P2a=0.01874, P2b=0.00149 |  |  |  |  |
|  |  |  | background ω | | ω0=0.0465, ω1=1.00000 |  |  |  |  |
|  |  |  |  | | ω2a=0.0465, ω2b=1.00000 |  |  |  |  |
|  |  |  | foreground ω | | ω0=0.0465, ω1=1.00000 |  |  |  |  |
|  |  |  |  | | ω2a=1.00000, ω2b=1.00000 |  |  |  |  |
